# Synthesis, microstructure, multifunctional properties of mayenite Ca_12_Al_14_O_33_ (C12A7) cement and graphene oxide (GO) composites

**DOI:** 10.1038/s41598-020-68073-4

**Published:** 2020-07-06

**Authors:** Chaval Sriwong, Chaiwat Phrompet, Wattana Tuichai, Attaphol Karaphun, Ken Kurosaki, Chesta Ruttanapun

**Affiliations:** 1 Center of Excellence in Smart Materials Research and Innovation, King Mongkut’s Institute of Technology Ladkrabang, Chalongkrung Road, Ladkrabang, Bangkok, 10520 Thailand; 2 Smart Materials Research and Innovation Unit, Faculty of Science, King Mongkut’s Institute of Technology Ladkrabang, Chalongkrung Road, Ladkrabang, Bangkok, 10520 Thailand; 3 Department of Physics, Faculty of Science, King Mongkut’s Institute of Technology Ladkrabang, Chalongkrung Road, Ladkrabang, Bangkok, 10520 Thailand; 4 Department of Chemistry, Faculty of Science, King Mongkut’s Institute of Technology Ladkrabang, Chalongkrung Road, Ladkrabang, Bangkok, 10520 Thailand; 5 Institute for Integrated Radiation and Nuclear Science, Kyoto University, 2, Asashiro-Nishi, Kumatori-cho, Sennan-gun, Osaka, 590-0494 Japan; 6 Thailand Center of Excellence in Physics, Ministry of Higher Education, Science, Research and Innovation, 328 Si Ayutthaya Road, Bangkok, 10400 Thailand

**Keywords:** Supercapacitors, Electronic properties and devices

## Abstract

The Pristine Mayenite Ca_12_Al_14_O_33_ (C12A7) Cement was simply synthesized by using solid-state reaction. The C12A7 and Graphene Oxide (GO) composites (C12A7_GO-x) with various contents of the GO suspension loading (x = 0 wt%, 1 wt%, 2 wt%, 3 wt%, and 4 wt%) were directly prepared by mixing the C12A7 and GO. X-ray diffraction results of pristine C12A7 and all C12A7_GO composites indicated a pure phase corresponding to the standard of C12A7 cement. Raman spectroscopy confirmed the existence of GO in all C12A7_GO samples. Scanning Electron Microscopy (SEM) showed the micrometer grain sizes and the occurrence of grain boundary interfaces for GO incorporation in all C12A7_GO samples. UV–Vis spectroscopy revealed the absorption value of all C12A7_GO samples and red shift near longer wavelengths when increasing the GO concentrations. The dielectric constant of C12A7_GO composites can be explained by the high density of free electron charges for the interfacial polarization on the GO surface. The maximum specific capacitance of C12A7_GO-4 electrode of 21.514 at a current density of 0.2 A g^−1^ can be attributed to the increase in the electrochemically active surface area for the formation of the electrical double layer capacitors behavior and the effects of high surface area GO connections. Also, the mechanical properties exhibited an increase in Vickers indenter hardness (*HV*) values with increasing GO contents. The highest *HV* value was 117.8 *H*V/2 kg at the C12A7_GO-4 sample. These results showed that the composite materials of the pristine C12A7 cement with GO were highly efficient. All in all, the GO material contained a high potential for enhancing low-cost cement materials in multifunctional properties such as optical, dielectric, electrochemical, and mechanical properties.

## Introduction

Owing to the extensive physical, chemical, and mechanical properties of the oxide ion, the conducting materials were produced by moving the oxide ions through the crystal structure. These materials have been widely studied and applied in various fields of technology for example superconductors, catalysts, ceramics, batteries, supercapacitors, solid oxide fuel cells, and photoelectric devise^1–5^. Among various kinds of the oxide ion conducting materials, calcium-alumina binary compound, 12CaO-7Al_2_O_3_ (12CaCO_3_-7Al_2_O_3_ or Ca_12_Al_14_O_33_, abbreviated as C12A7) cement, is a kind of mineral mayenite structure^6–10^. The C12A7 complex oxide material is widely used in calcium aluminate cement^6–9^. At room temperature, bulk C12A7 material has a cubic body centered unit cell within the I-43d space group and an inner diameter of ~ 0.4 nm. Normally, the crystal structure of bulk C12A7 material is represented by the formula [Ca_24_Al_28_O_64_]^4^^+^·2O^2−^ (C12A7:O^2−^)^6,9,11^ and the positively charged ion (cation) [Ca_24_Al_28_O_64_]^4+^ makes up the framework of equivalent 12 sub-nanometer-sized cages. Two extra-oxygen anions (O^2–^) randomly occupy 2 out of 12 cages in the crystal structure of bulk C12A7 material as one cage is positively charged with a nominal charge of + (1/3) |e| on average and another one is the extra-framework O^2–^ anions working as counter anions to maintain charge neutrality^6,9,11–14^. In addition, the encaged O^2–^ anions are loosely bound to the positively charged cages and can be substituted by other anions with an appropriate size, including unconventional ionic species such as O, O_2_^–^, O_2_^2–^, H^–^, and OH^–^^14–19^. Consequently, the electronic conduction mechanism at room temperature can be switched from hopping to band conduction with the conductivity changes from < 10^–10^ S/cm to 1.5 × 10^3^ S/cm^20^.

In previous reports, Rudradawong and Ruttanapun^21^ described that the electrical conductivity of a polycrystalline C12A7 was prepared using a solid state reaction, increased by approximately 10^9^ orders of magnitude at room temperature when experiencing Mg heat treatment for 10 h with the highest electrical conductivity of 7.65 S/cm at 573 K and low activation energy of 0.038 eV. Ruttanapun et al.^22^ investigated the effect of Fe^3+^-doped Ca_12_Al_14_O_33_ cement on optical and thermal properties Fe ions doped C12A7 were prepared through a solid-state reaction in atmosphere oxygen. It was reported that the substitution of Fe^3+^ into Al^3+^ sites of C12A7 cement directly affected both its optical gap and thermal conductivity. Similarly, the excitation of free electrons in the C12A7 cement was indicated by absorption spectra at 2.8 eV with an optical energy gap of 3.5 eV as reported in Phrompet., et al.’s study^23^, which is consistent with the first-principles calculations of the band energy level. Moreover, Rudradawong et al.^24^ reported that a positive ion conduction of Mayenite C12A7/nano-carbon black composites (C12A7/nCB) resulted from oxygen ion vacancy occupying in extra framework which caused enhanced dielectric constant, Seebeck coefficient, electrical conductivity, and reduced thermal conductivity.According to several studies, bulk C12A7 material can be modified in terms of electrical conductivity, optical properties, and flexural and compressive strength by substituting the electron into the extra framework O^2–^ anions in the nano-cages using a modified graphene based material (graphene oxides (GO))^25–28^. Most of the recent studies on GO materials have been known for enhancing other favorable properties such as high carrier mobility (> 15,000 cm^2^/V s at carrier densities of 1,013 cm^−2^), large spring constants (1–5 N/m), and correspondingly improved exceptional Young modulus values (> 0.5 to 1 TPa)^29,30^. According to Karim et al.’s study^31^ , the C12A7 is modified by the preparation of a conductive nano- caged C12A7 particles using 2D nanostructure GO composite.Subsequently, Yakovlev et al.^32^ indicated that the enhanced electrical conductivity of electride C12A7 (C12A7:e^−^) nanoparticles was successfully prepared via a carbon nano-reaction process with reduced grapheme oxide (rGO) composite coated by nano-caged C12A7 particles. In another study, Velez et al.^27^ have studied the bulk mechanical properties of C12A7. Their study displayed an elastic moduli tensor and the calculated Young’s modulus was 138.7 GPa which was in good agreement with the value obtained from nano-indentation. Hosono et al.^6^ have revealed the mechanism of the oxygen ions conduction in C12A7 cement as mainly being controlled by the diffusion of free O^2−^ and O^−^ ions. Dimov et al.^28^ have reported the composited nano-engineered cement with GO provided an ultrahigh strength. Li et al.^33^ have reported the improving flexural and compressive strength of GO/C12A7 composite materials for the repair material applied in the construction field. Thus, the GO material shows a good performance with the C12A7cement materials which are improved by the composition of C12A7 particles and 2D nanostructure GO (as presented by C12A7_GO). However, there are a few researches on the improvement of multifunctional properties investigation regarding the composite C12A7_GO materials.

Herein, this study investigates the multifunctional properties of optical, dielectric, electrochemical, and mechanical properties with a focus on C12A7_GO composite materials. The study aims to investigate the composite materials of the pristine C12A7 cement with GO material through a sample mixing method. In the process of preparation, various contents of GO suspension loading (0, 1, 2, 3, and 4 wt%) in the pristine C12A7 cement were considered as a microstructure, and a potentiality for multifunctional properties (optical, dielectric, electrochemical, and mechanical properties) due to the free electrons interaction between GO surfaces and free extra framework O^2–^ anions in C12A7 lattice.The pristine C12A7 and all C12A7_GO composite materials were investigated in terms of phase formation, microstructure, optical, dielectric, electrochemical and mechanical properties.

## Results and discussion

### XRD analysis

**Figure 1:**
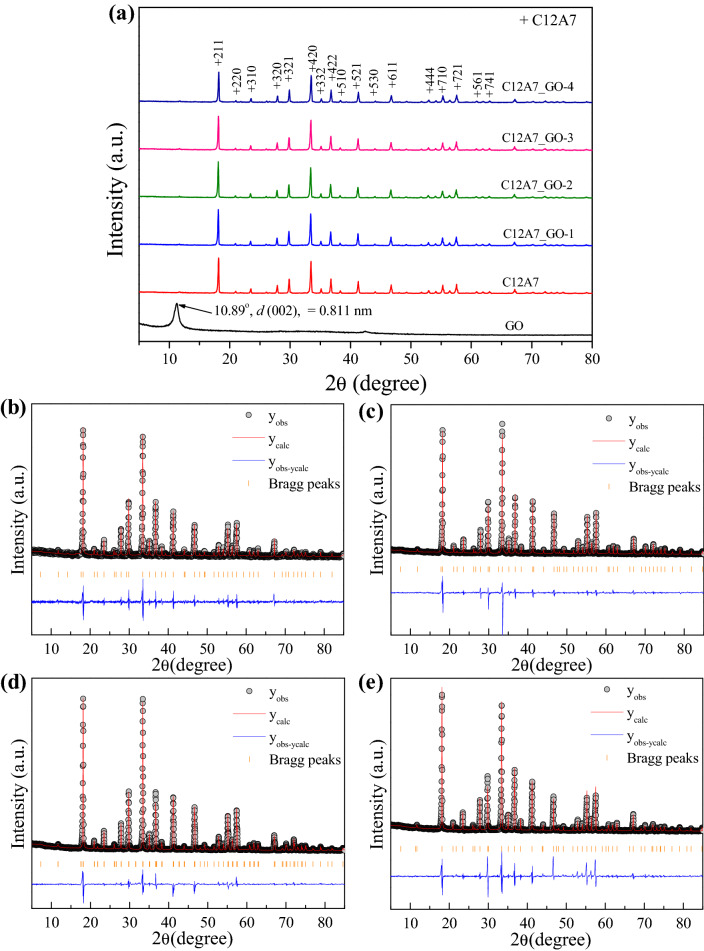
(**a**) XRD patterns and (**b**–**e**) Rietveld profile fits of the pristine C12A7 and C12A7_GO-1, C12A7_GO-2, and C12A7_GO-3 composite samples.

The XRD patterns of the GO, pristine C12A7, and all C12A7_GO samples are shown in Fig. 1a. The Rietveld profile fitting of the pristine C12A7, and C12A7_GO-1, C12A7_GO-2, and C12A7_GO-3 samples are shown in Fig. 1b–e. As seen in Fig. 1a, the characteristic diffraction peak of GO is appeared at 2θ = 10.890°, which was well indexed to the (002) plane and presented the layer distance (d-spacing) of 0.811 nm, corresponding to the standard GO pattern^26^. In line with Dikin et al.’s study^35^, the results indicated a layer distance of approximately 0.8 nm showing one molecule-thick layer of water and hydrogen-bonded between the layer GO sheets. Furthermore, the edges of GO sheets consisted of hydrophilic oxygenated graphene sheets supporting the existing oxygen functional groups, which is vital for the pristine C12A7 cement composite. Moreover, the C12A7_GO samples under all different conditions showed that all of the main diffraction peaks from 2θ = 18.0°–70.0° corresponded to (211), (310), (320), (321), (420), (332), (422), (551), (611), (444), (640), (642), and (831) planes as indexing the pristine Ca_12_Al_14_O_33_ cement phase of cubic structure with space group of *I-43d* in the standard data of JCPDS:00-009-0413^36^. The intensity peak of the C12A7_GO samples was decreased due to the strong continuous network of GO sheets with the agglomeration and over stacking on C12A7 surface, which is consistent with the reported values in the literature^31^. In addition, lattice parameters and fitting parameters of the samples were calculated by using the Rietveld profile fits of the pristine C12A7, C12A7_GO-1, C12A7_GO-2, and C12A7_GO-3 samples as summarized in Table 1 and displayed in Fig. 1b–e. Figure 1b–e displays the fitting refinement and calculation lattice parameters of the samples. The results were in a good agreement with the standard data. The calculated lattice parameters along *a*-axis were increased with increasing GO content as 11.975(4)**,** 12.009(8)**,** 11.993(3), 12.020(5) and 12.004(4) Å for the pristine C12A7, C12A7_GO-1, C12A7_GO-2, C12A7_GO-3 and C12A7_GO-4 samples, respectively. It might be the result of free electrons interaction between GO surfaces and free extra framework O^2−^ anions in C12A7 lattice. The crystallite size (D) of the pristine C12A7 and all C12A7_GO samples was calculated using the diffraction peaks and planes by Scherer’s Eq. (1): [37]

1 $${\text{D}} = {\text{k}}\lambda /\beta {\cos}\theta$$

where D is the crystallite size, λ is the X-ray wavelength, k is the shape factor of value 0.9, θ is the diffraction angle, and β is the full width at half maximum. All calculated D values of the pristine C12A7 and all C12A7_GO samples are listed in Table 1. The obtained D value was 58.45 nm for the pristine C12A7 sample. The composite samples of GO loading C12A7 were increased with increasing GO content as 63.52, 63.85, 66.12 and 68.78 nm for C12A7_GO-1, C12A7_GO-2, C12A7_GO-3 and C12A7_GO-4 samples, respectively.

**Table 1:** Lattice parameters, the obtained parameter of fitting were calculated using the Rietveld profile fits and the crystallite size (D) of the pristine C12A7 and C12A7_GO-1, C12A7_GO-2, and C12A7_GO-3 composite samples.

Sample	Lattice parameters (Å)	R brag	R profile	Weight profile	Goodness of fitting	D (nm)
Pristine C12A7	11.975 (4)	5.1093	23.7010	29.0889	4.0114	55.45
C12A7_GO-1	12.009 (8)	4.8815	16.3390	20.7068	2.3551	63.52
C12A7_GO-2	11.993 (3)	4.9113	17.6736	21.8963	2.4875	63.85
C12A7_GO-3	12.020 (5)	4.9119	15.8569	19.4161	2.0935	66.12
C12A7_GO-4	12.004 (4)	5.0943	11.1953	16.0558	3.6428	69.78

### Raman spectra analysis

**Figure 2:**
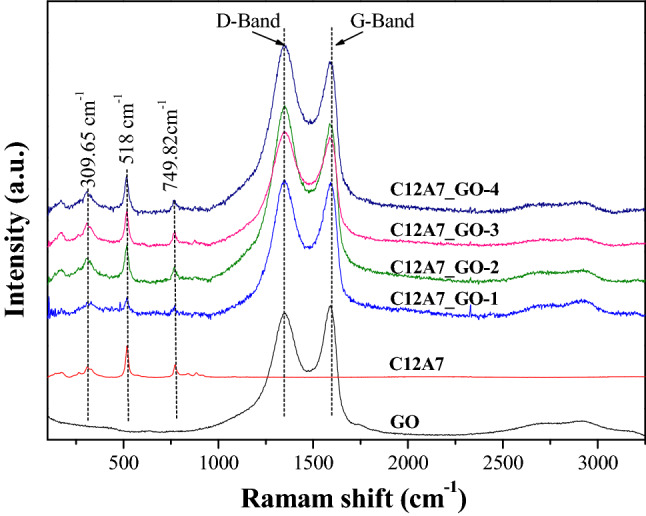
Raman spectra of the pristine C12A7 and all C12A7_GO composite samples**.**

Figure 2 shows the Raman spectra of the GO, pristine C12A7 and all C12A7_GO samples. The main peaks of pristine C12A7 phase were observed at the Raman shift region of 100–1,000 cm^–1^, which is attributed to the lattice framework of the Ca_12_Al_14_O_33_ cement structures. The Raman peak at around 309.65 cm^–1^ was caused by the vibrations of oxygen (O^2–^) framework in Ca[AlO_4_] crystalline and Ca–O bonding^25^. Next, the two maim Raman peaks at around 518.0 and 749.82 cm^–1^ indicate the vibrations of symmetric stretching (ν1) of the Al–O and Al–O–Al linkage bonds, respectively, in the lattice structure of AlO_4_^–5^ tetrahedra ^25^.When the content loading of GO in C12A7_GO is increased, the Raman shift peak of the samples can be observed in two main peaks at around 1,338 and 1586 cm^–1^. The first peak (1,338 cm^–1^) corresponds to the hexagonal graphitic layers of D-band, which is related to the defects and disorder. The second peak (1586 cm^–1^) corresponds to the G-band, which related to sp^2^-bonded carbon atom vibration in 2D hexagonal lattice owing to the strong continuous network of GO sheets^26^. Furthermore, the occurrence peaks of 2D and D + G band at around 2,688.83 and 2,942.56 cm^−1^, respectively, can be confirmed by the second order Raman spectra. It is forming an orientation, stacking defects, and a defect-induced double resonance of inter-valley scattering process during the composites. Interestingly, the obtained composite phases between pristine C12A7 phases and GO sheets without other phases were detected, which can strongly confirm the C12A7_GO composites by XRD technique. Therefore, the above results confirm that the complete composite system of the C12A7 cement and GO composite structure was due to the existence of free extra framework of O^2-^ in C12A7 structure presented by O^2-^ vibrations mode.

### FTIR analysis

**Figure 3:**
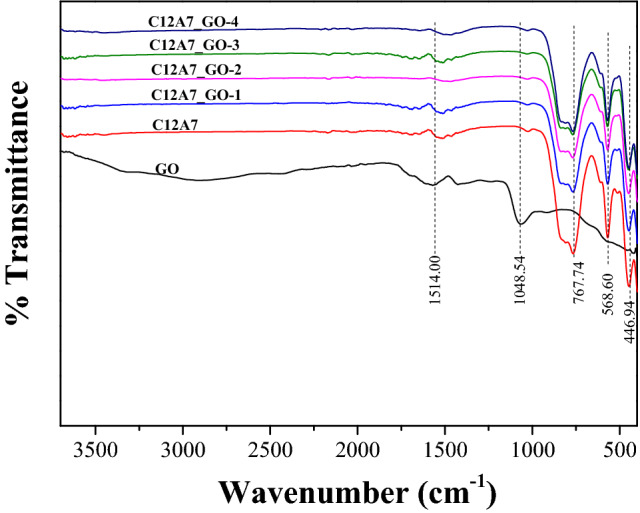
FTIR spectra of GO, the pristine C12A7 and all C12A7_GO composite samples**.**

FTIR measurements were used to study the GO, the pristine C12A7 and all C12A7_GO samples as shown in Fig. 3. As can be seen in Fig. 3, the vibration bands of the GO sample was appeared at approximately 3,500–2,500 cm^−1^, 1514.00 cm^−1^, and 1,048.54 cm^−1^, corresponding to the presence of –O–H stretching of hydroxyl, –C=O stretching of carbonyl/carboxyl, and –C–O–C– stretching of epoxy groups, respectively^31,38^. Regarding all samples of Go loading C12A7 cement, the peak at approximately 790 cm^−1^ can be associated with the stretching mode of the free oxygen ions (O^2−^) in extra framework of the CAO structure^39^. Moreover, both vibration peaks at approximately 568 and 449.94 cm^−1^ are attributed to the stretching mode of Ca, Al and O atoms (Ca–O–Al stretching) as the characterization of insulating Ca_12_Al_14_O_33_ cement^39^. These results can confirm the pristine C12A7 phase, corresponding to XRD results and Raman spectra.

### TGA and DTA diagrams analysis

**Figure 4:**
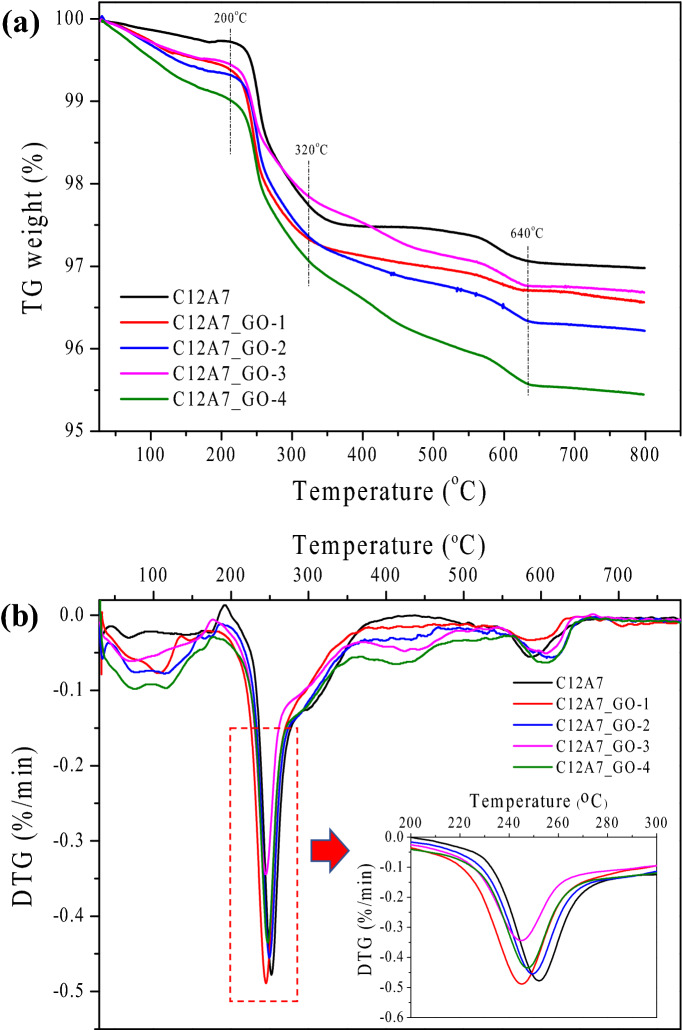
(**a**) TGA curve, (**b**) DTG curve of the pristine C12A7 and all C12A7_GO samples.

Figure 4a,b shows the TGA and DTG diagrams analysis which was done to study the behavior and weight loss of the pristine C12A7 and all C12A7_GO samples. As seen in Fig. 4a, the first phase of the weight loss of all samples at the temperature of 200 °C slightly decreased due to the removal of physically absorbed water on the surface of Ca_12_Al_14_O_33_ cement. The second phase of weight loss at the temperature ranging from 200 to 320 °C was assigned to the mass loss of the decomposition and/or oxidation process of water, Al_2_O_3_ and CaCO_3_ structures which lead to the formation of Ca_3_Al_2_(OH)_12_cement^40,41^. In the next phase of TGA curve, the mass loss temperature ranging from 320 to 640 °C was mainly attributed to the decomposition of Ca_3_Al_2_(OH)_12_ cement co-occurring with a transformation phase to Ca_3_Al_2_(OH)_6_ cement. Finally, in the fourth phase of the weight loss, the decomposition of Ca_3_Al_2_(OH)_6_ cement, which is the transformation phase to Ca_12_Al_14_O_33_ cement, was observed at higher temperature of 640 ^o^ C. In addition, the weight loss of all samples with GO loading C12A7 composites was higher than that of the pristine C12A7 cement. In Fig. 4b, the DTG curve was shifted to lower temperatures with increasing the GO content. This result showed that an improvement of the thermal and phase transformation was due to C12A7 cement content and GO sheets composites.

### BET analysis

**Figure 5:**
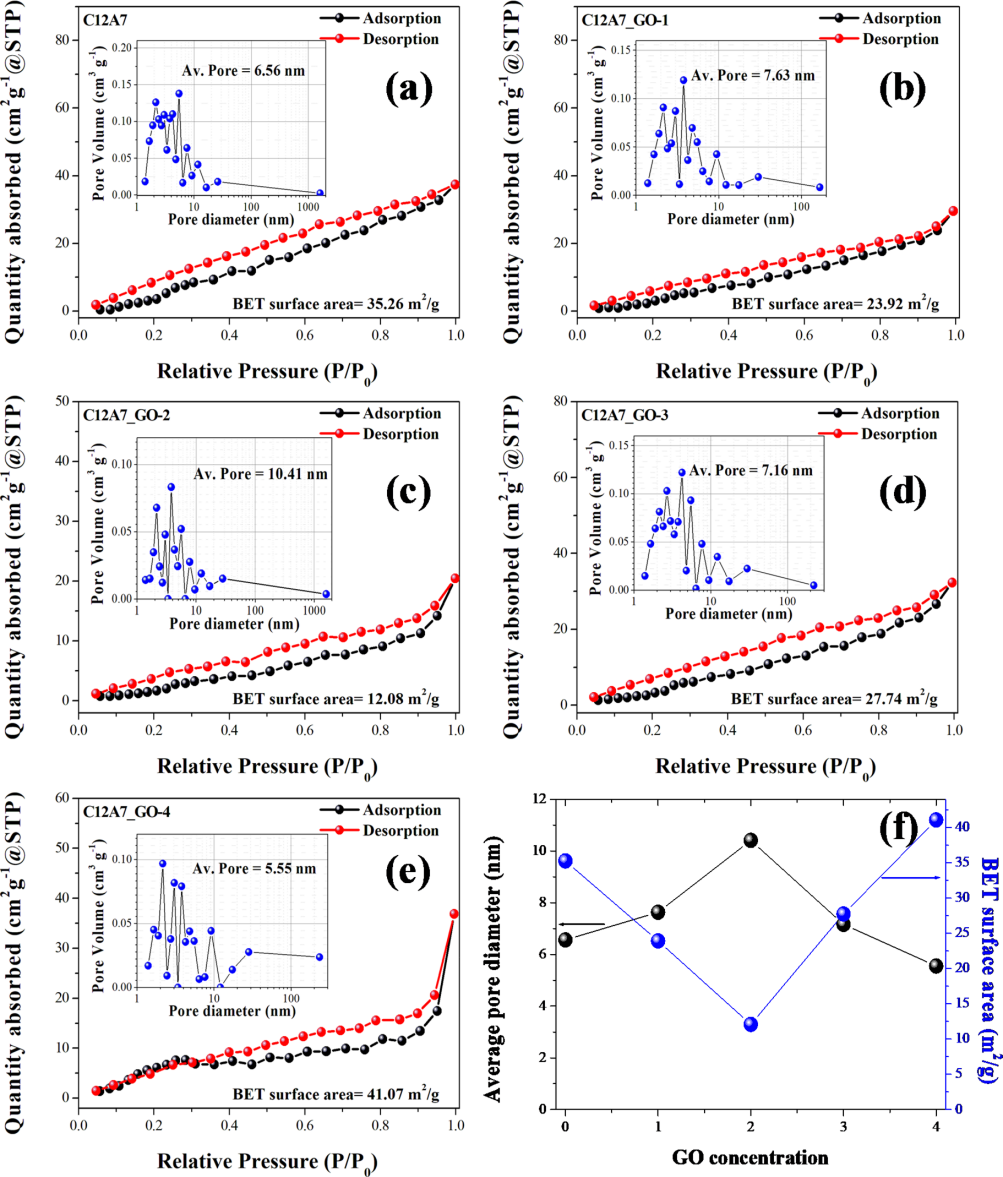
(**a**–**e**) Nitrogen adsorption isotherms with inset pore volume curves of the pristine C12A7 and all C12A7_GO samples, and (**f**) the plots of average pore sizes and BET surface area as a function of GO concentration.

Figure 5a–f show Nitrogen adsorption isotherms with inset pore volume curves of the pristine C12A7 and all C12A7_GO samples, the plots of average pore sizes, and specific BET surface area as a function of GO concentration, respectively. As seen in Fig. 5a–e, the adsorption/desorption Nitrogen isotherms exhibited a typical type-IV isotherm, suggesting the characteristic of mesoporous structure in particles. Moreover, the specific BET surface area of the pristine C12A7 and all C12A7_GO samples was estimated from the area of a closed curve as shown in Fig. 5f, which found to be 35.26 for the pristine C12A7 and C12A7_GO-1, C12A7_GO-2, C12A7_GO-3 and C12A7_GO-4 samples of 23.92, 12.08, 27.74 and 41.07 m^2^/g, respectively. Additionally, the average pore sizes of the pristine C12A7 and all C12A7_GO were calculated by using BJG technique as shown the inset Fig. 5a–e. The results were found to be 6.56, 7.63, 10.41, 7.16 and 5.55 nm for the pristine C12A7, C12A7_GO-1, C12A7_GO-2, C12A7_GO-3 and C12A7_GO-4 samples, respectively. The maximum specific BET surface area was 41.07 m^2^/g of C12A7_GO-4 samples. The effect was due to the surface C12A7 cement being uniformly decorated by the network structure of GO sheet, which is promoting the surface area^42^.

### SEM and EDS analysis

**Figure 6:**
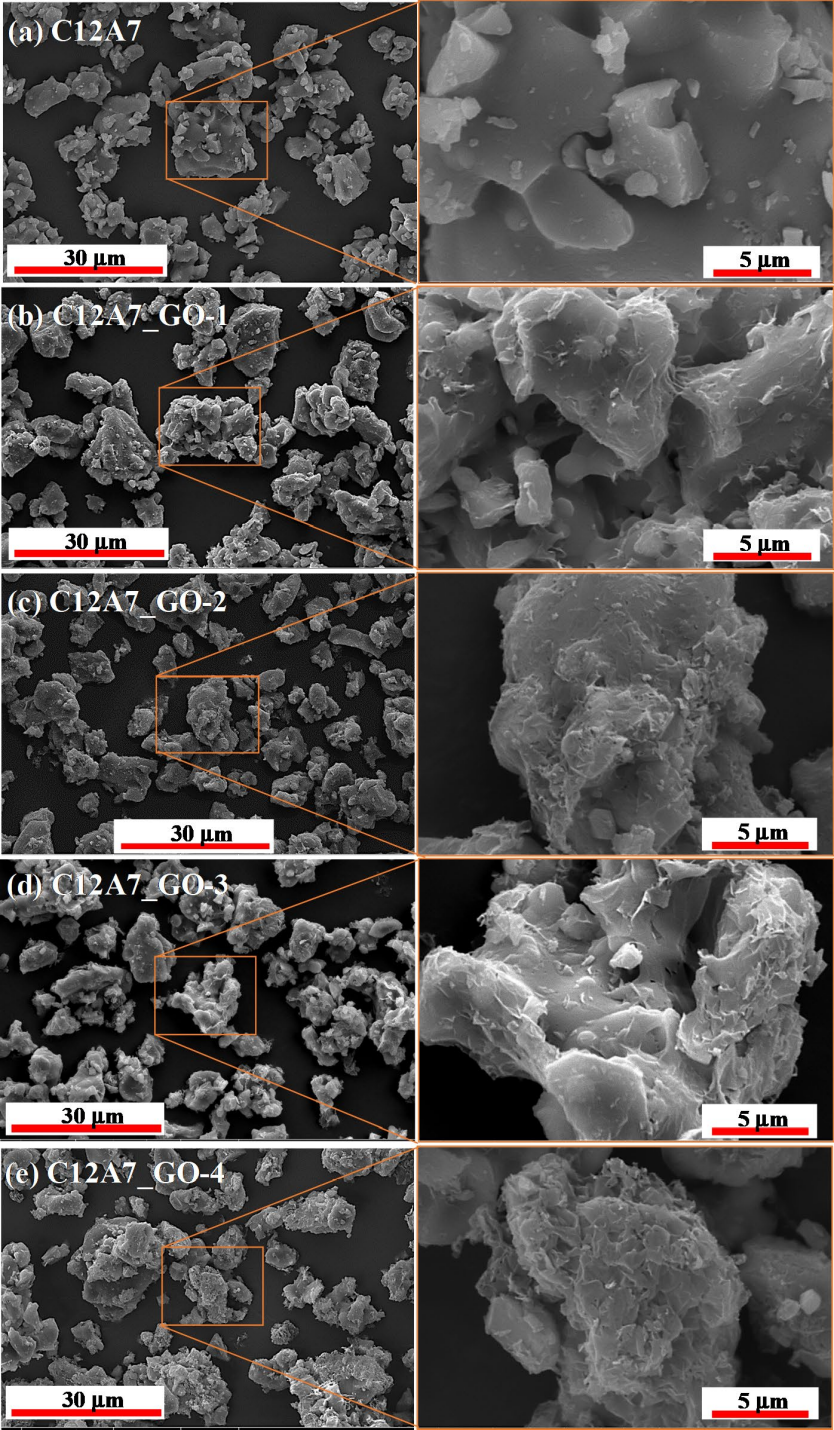
(**a**–**e**) SEM images with inset being a high magnification view of selected area on surface of the pristine C12A7 and all C12A7_GO composite samples.

**Figure 7:**
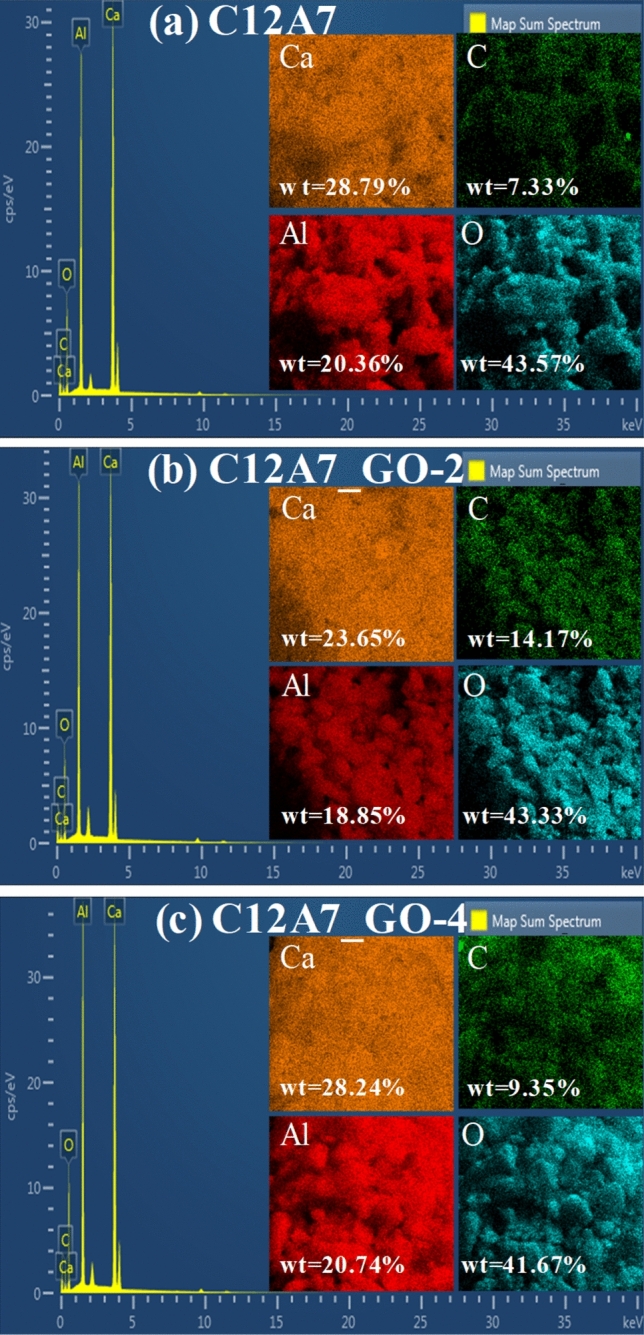
(**a**–**c**) EDS spectra and mapping images for Al, Ca, C and O elements of the pristine C12A7, C12A7_GO-2 and C12A7_GO-4 composite samples, respectively.

Figure 6a–e show SEM images with inset being a high magnification view of selected area on the surface of pristine C12A7 and all C12A7_GO samples. It was clear in Fig. 6a–e that the similar particles of C12A7 with irregular edges had micrometer grain sizes in all samples. Furthermore, all composite samples of GO loading displayed uniform decoration and covering by GO thin sheets on the surface of the pristine C12A7 cement. The results confirmed that the GO sheets can exist and they can be decorated and incorporated with pristine C12A7 cement. This resulted in H-bonding interaction between hydrogen atoms of oxygen functional groups, carboxylic and hydroxyl (–OH) on the skeleton of GO structure, and oxygen atoms of the C12A7 structure. This obtained result was influenced by the mixing method used for acetone media as it enhanced the incorporation and occurrence of the grain boundary interfaces inC12A7_GO composite cements. The results showed a high magnification view inset in Fig. 6b–e. It should be mentioned that the surface of C12A7_GO composite cements appeared to increase GO sheet particles on the C12A7 microparticles surface along with increasing the GO loading contents. The EDS results of the pristine C12A7, C12A7_GO-2 and C12A7_GO-4 samples are shown in Fig. 7a–c. The results presented the Ca, Al, O and C atoms in these samples indicating that all samples formed the Ca_12_Al_14_O_33_ cement phase structure. There also was a homogeneous distribution of the Ca, Al, O and C atoms on the powder surfaces.

### UV–Vis spectroscopy analysis

**Figure 8:**
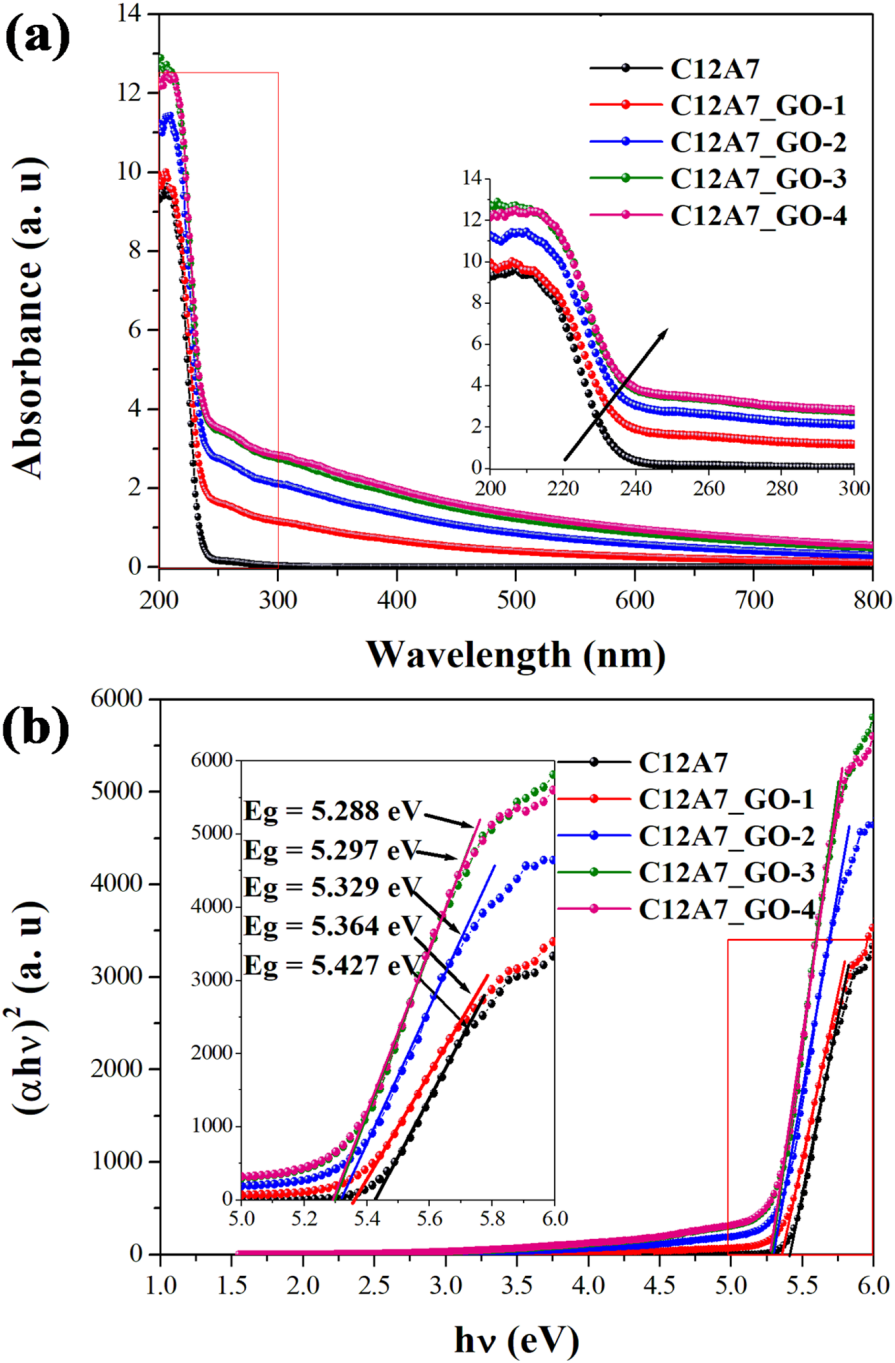
(**a**) UV–Vis spectra and (**b**) plots of (αhν)^2^ versus photon energy regarding the optical band gap determination of the pristine C12A7 and all C12A7_GO composite samples, respectively.

Figure 8a shows the UV–Vis absorption spectra of the pristine C12A7 and all C12A7_GO samples. The absorption edge of the C12A7 sample appeared at approximately 210.01 nm with an observed a red shift near longer wavelengths at 211.54, 215.72, 218.41 and 218.50 nm in the GO loading C12A7 samples of C12A7_GO-1, C12A7_GO-2, C12A7_GO-3 and C12A7_GO-4, respectively. Similar to the studies conducted by Matsuishi et al.^8^ Rudradawong et al.^21^ and Rudradawong et al.^24^ the result was due to the transition of free electrons from the extra O^2–^ states inside a cage to the CCB. The optical band gaps (E_g_) of the pristine C12A7 and all C12A7_GO samples were determined by fitting the absorbance data in Fig. 8a to Eq. (2):

2 $$\alpha h\nu = E_{D} (h\nu - E_{g} )^{1/2}$$

where E_g_ is the direct band gap, hν is the photon energy, α is the optical absorption coefficient, and E_D_ is a constant. The E_g_ values of the pristine C12A7 and all C12A7_GO samples were obtained by extrapolating the linear regions of these plots to zero absorption, as shown in Fig. 8b. As seen in this Table, the E_g_ value of the pristine C12A7 sample was 5.427 eV and that of samples with GO loading C12A7 was decreased to 5.364, 5.329, 5.297 and 5.288 eV for C12A7_GO-1, C12A7_GO-2, C12A7_GO-3 and C12A7_GO-4, respectively. The E_g_ values were in a good agreement with the results of Hayashi et al.’s study^43^, where it was determined by the chemical identity of the extra framework species. It can be varied in the range of ∼4−6 eV. Also, the decrease of E_g_ in GO loading C12A7 samples as a result of the red shift phenomenon in the UV absorption spectra was suggested to be originated from the formation of free electrons interaction between free extra framework O^2–^ anions and GO surfaces in C12A7_GO lattices. Furthermore, the transition of free electron movement in the strong continuous network of GO sheets was supported by the absorption energy at the surface of the materials, according to the Hara et al.’s report.^44^.

### Dielectric properties

**Figure 9:**
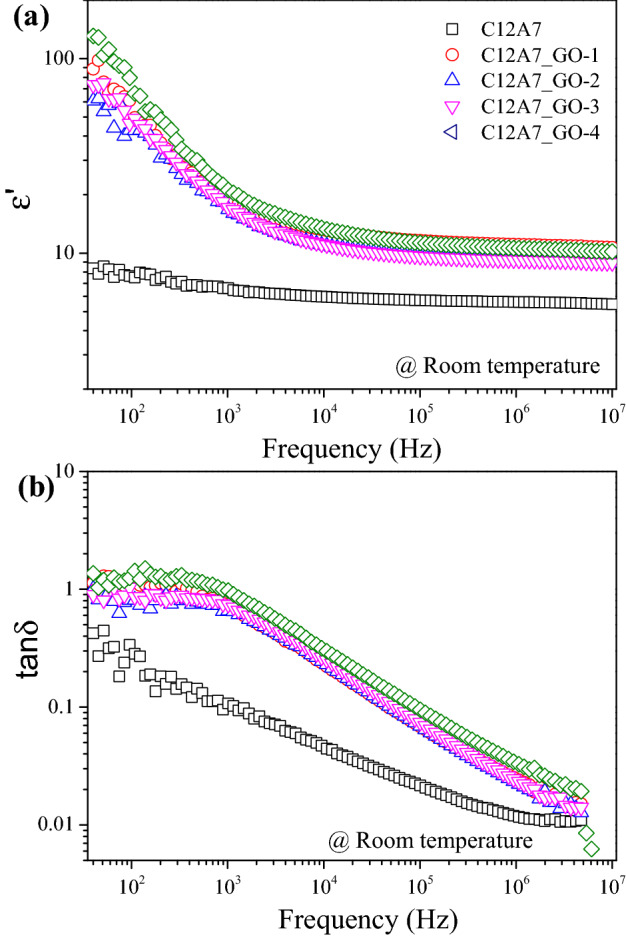
The frequency dependence of ε′ (**a**) and tanδ (**b**) of the pristine C12A7 and all C12A7_GO composite samples at room temperature.

The dielectric behavior of the pristine C12A7 and all C12A7_GO samples at room temperature is shown in Fig. 9a,b. Figure 9a indicates that the εʹ value increases with increasing GO concentration. The εʹ value of the pristine C12A7, C12A7_GO-1, C12A7_GO-2, C12A7_GO-3 and C12A7_GO-4 samples was determined at 1 kHz and found to be 6.61, 19.44, 16.80, 17.14 and 21.11, whereas tanδ value was found to be 0.107, 0.715, 0.658, 0.740 and 0.974, respectively, as shown in Fig. 9b. These results can be explained by the internal of filler having many free charges on the surface of C12A7_GO samples, which mainly affects the interfacial polarization^45–47^. Theoretically, interfacial polarization can be effective at low frequency^46^. In addition, the GO sheets were agglomerating and over stacking on C12A7 surface, which increase the accompanying micro capacitor. Moreover, the results were highly effective in increasing the dipolar polarization at the interfacial region of C12A7_GO samples^45^.

### Electrochemical properties

**Figure 10:**
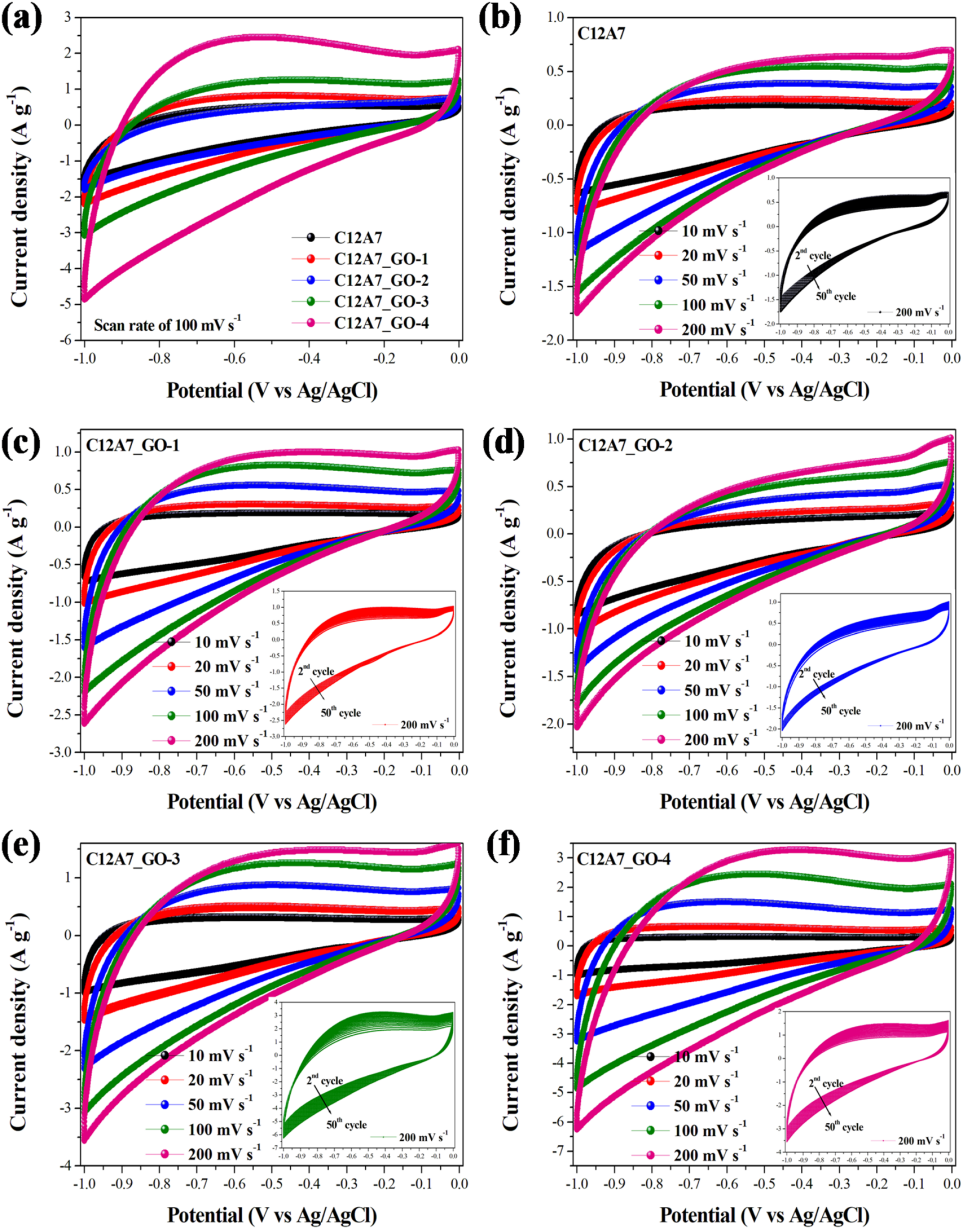
(**a**) CV curves at a scan rate of 100 mV s^−1^ of the pristine C12A7 and all C12A7_GO electrodes. (**b**–**f**) CV curves at different scan rates with inset cycling stability using CV test at a scan rate of 200 mV s^−1^ of the pristine C12A7 and all C12A7_GO electrodes, respectively.

Figure 10a shows CV curves of the pristine C12A7 and all C12A7_GO electrodes in 1.0 to a − 0.0 V voltage window at a scan rate of 100 mV s^−1^. Figure 10b–f show CV curves of the pristine C12A7 and all C12A7_GO electrodes in various scan rates with inset cycling stability using CV test at a scan rate of 200 mV s^−1^. It is obviously seen in Fig. 10a that the CV curve of the pristine C12A7 had poor capacitive properties, whereas all C12A7_GO electrodes were similar in showing an ideal electrochemical double layer capacitor (EDLC) behavior, which was rectangle shaped with increasing GO content^48,49^. Additionally, Fig. 10b–f show the CV curve remaining quasi-rectangular with increased scan rate indicating good capacitive properties for all C12A7_GO electrodes. Also, the response current was increased with an increase in scan rate suggesting an excellent rate capability of the pristine C12A7 electrode with adding GO concentrations. It can be seen in the inset of Fig. 10b–f that the characterization of CV curve was increased with more cycling test due to the typical pattern of electrochemically GO behavior^48–50,53^.

**Figure 11:**
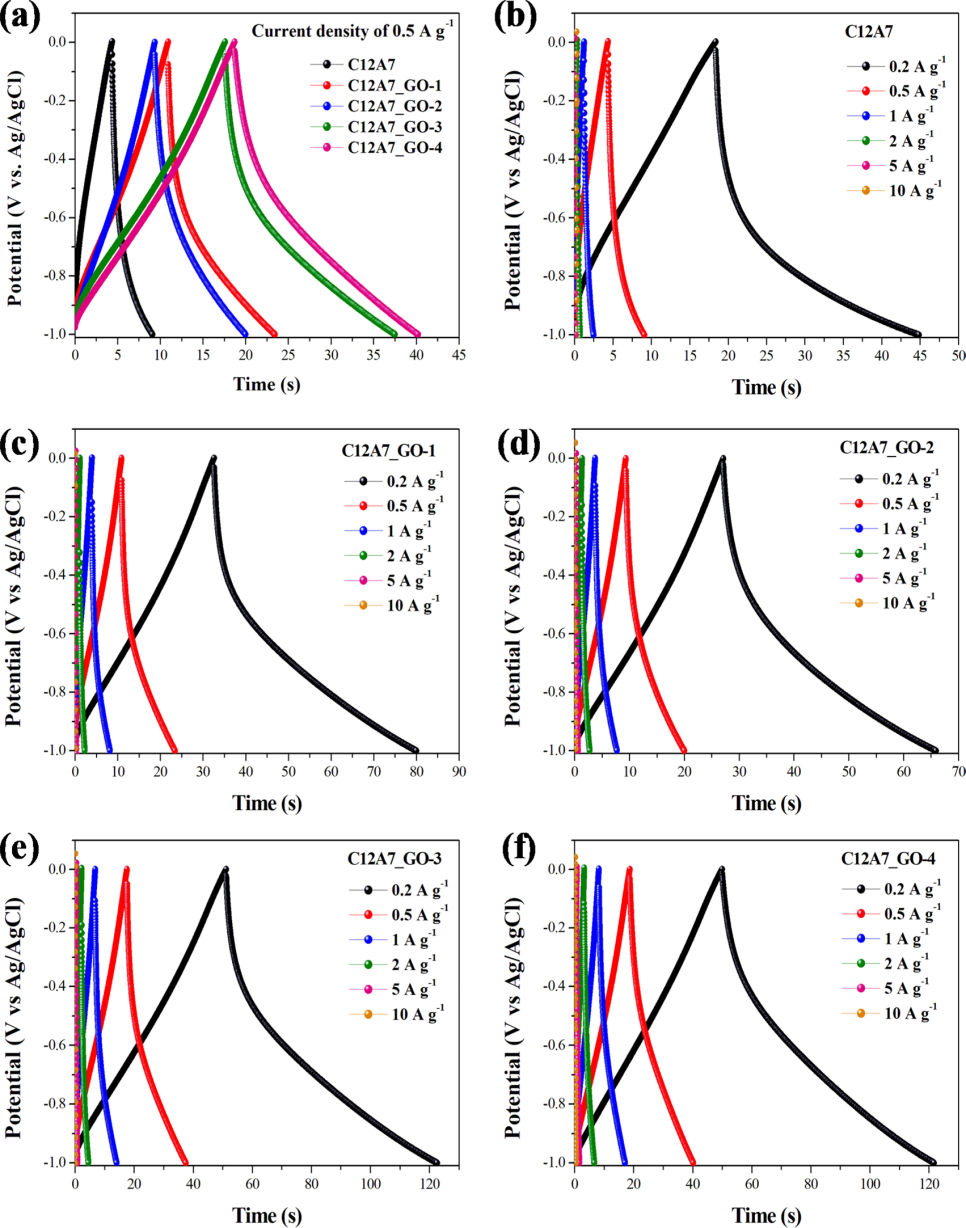
(**a**) GCD curves at a current density of 0.5 A g^−1^ of the pristine C12A7 and all C12A7_GO electrodes. (**b**–**f**) GCD curves at different current densities of the pristine C12A7 and all C12A7_GO electrodes, respectively.

**Figure 12:**
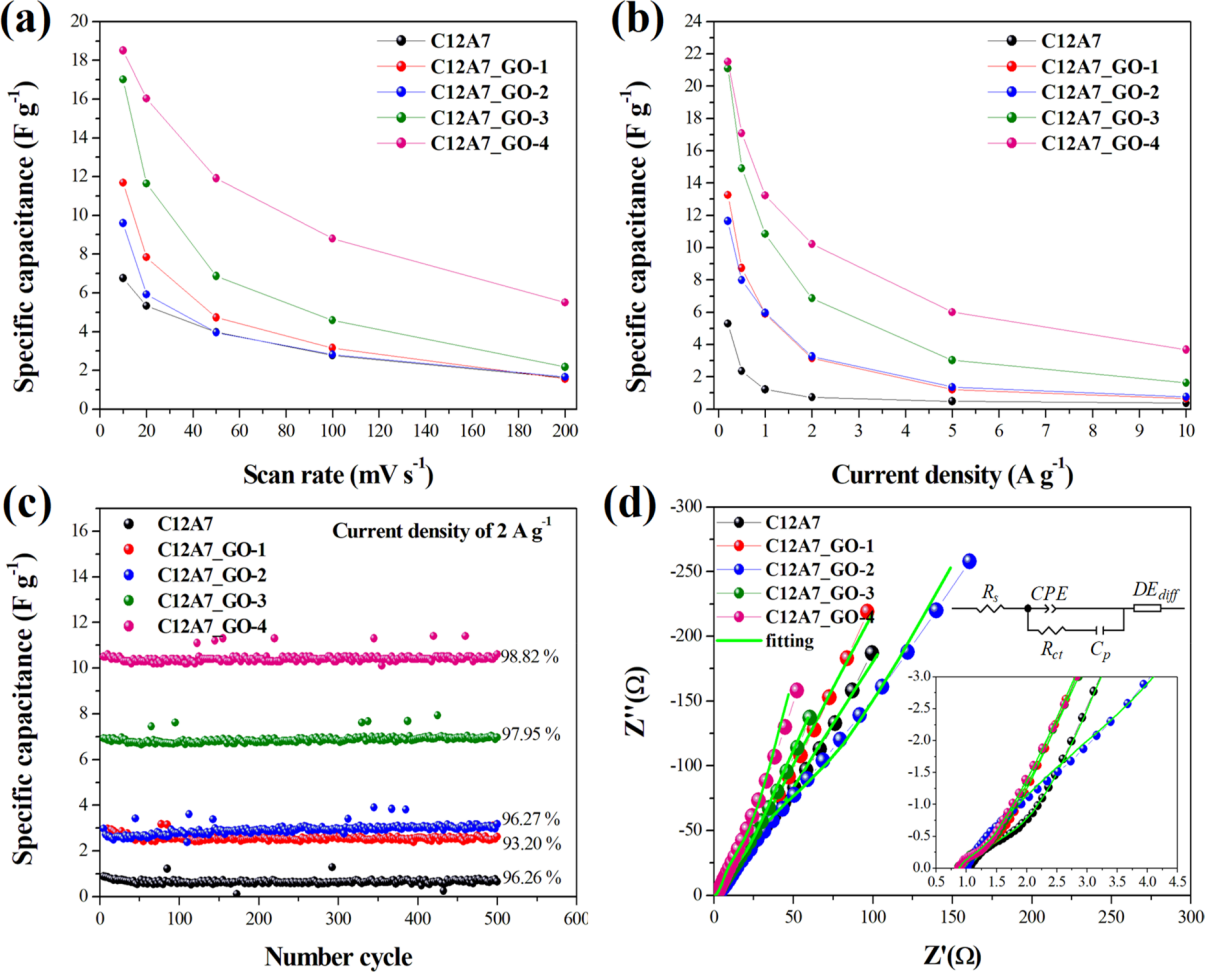
(**a**,**b**) Specific capacitance plots of the pristine C12A7 and all C12A7_GO electrodes at different scan rates and current densities were calculated by CV and GCD tests, respectively. (**c**) Cycling stability of the pristine C12A7 and all C12A7_GO electrodes using GCD test at a current density of 2 A g^−1^. (**d**) Fitting Niquist impedance plots with inset and the enlargement of the plots near origin and an equivalent circuit for fitting the pristine C12A7 and all C12A7_GO electrodes.

Figure 11a–f show approximately triangle-shaped curves suggesting capacitors with high reversibility and ideal capacitor behaviors^48–50,53^. Figure 11a indicates GCD curves of the pristine C12A7 and all C12A7_GO electrodes in a − 1.0 to 0.0 V voltage windows at a current density of 0.5 A g^−1^. Figure 11b–f show different current densities of the pristine C12A7 and all C12A7_GO electrodes in 0.2–10 a g^−1^. These features mainly originated from the electric double layer at the surface electrodes due to the interface among 1 M KOH aqueous electrolyte interface and the pristine C12A7 and all C12A7_GO electrodes. The specific capacitance of the pristine C12A7, C12A7_GO-1, C12A7_GO-2, C12A7_GO-3 and C12A7_GO-4 electrodes was calculated from the integral area of closed CV curves using the following Eq. (3):

3 $$C_{sc} = \int {idv} /2m \, v \, \Delta V$$

where *C*_*sc*_ is the specific capacitance (F g^−1^), *i* is the measured current (A), *v* is scan rate, *m* is the mass of active materials in each electrode (g), and *ΔV* is the total potential deviation (*V*), respectively. Figure 12a shows the *C*_*cs*_ value as found to be 6.764, 11.686, 9.604, 17.006, and 18.512 F g^−1^ at 10 mV s^−1^ for the pristine C12A7, C12A7_GO-1, C12A7_GO-2, C12A7_GO-3 and C12A7_GO-4 electrodes, respectively. The *C*_*cs*_ values were estimated from the obtained GCD curves using the following Eq. (4):

4 $$C_{sc} = i/m(\Delta V/\Delta t)$$

where *i* is the discharge current density (A g^−1^), *ΔV/Δt* is the slope of discharge curves after the R_i_ drop, respectively. In Fig. 12b, it can be seen that the *C*_*sc*_ values decreased with increasing current density. The *C*_*sc*_ values were found to be 5.291, 13.259, 11.646, 21.100, and 21.514 F g^−1^ at a current density of 0.2 A g^−1^ for the pristine C12A7, C12A7_GO-1, C12A7_GO-2, C12A7_GO-3 and C12A7_GO-4 electrodes, respectively. These *C*_*sc*_ values of the pristine C12A7 and all C12A7_GO electrodes are summarized in Table 2. The excellent electrochemical properties were obtained from C12A7_GO-4 sample as a promising electrode active material for EDLC. Figure 12c shows the capacitance retention rate of the pristine C12A7 and all C12A7_GO electrodes at a current density of 2 A g^−1^. The results indicated that the enhanced activity of the composite samples may be due to an enrichment effect via diffusion within the modified electrode and highly useful GO surface area. Also, the free electron charge was transferred to the free extra framework O^2–^ anions and GO surfaces in C12A7_GO lattices^26^. The C12A7_GO-4 electrode retained 98.82% in 1 M KOH aqueous electrolyte after 1,000 cycles, which is an excellent cycle stability indicating stable energy-storage processes during long cycle charging and discharging^51^.

**Table 2:** The *C*_*sc*_ values at different scan rates and current densities were calculated by CV and GCD test, respectively, and EIS analysis of the pristine C12A7 and all C12A7_GO electrodes is shown.

Parameters	Electrodes				
	C12A7	C12A7_GO-1	C12A7_GO-2	C12A7_GO-3	C12A7_GO-4
C_sc_ (F g^−1^) by CV					
10 mV s^−1^	6.764	11.686	9.604	17.006	18.512
20 mV s^−1^	5.330	7.837	5.926	11.639	16.024
50 mV s^−1^	3.974	4.727	3.964	6.872	11.917
100 mV s^−1^	2.781	3.157	2.818	4.580	8.804
200 mV s^−1^	1.618	1.568	1.660	2.173	5.508
C_sc_ (F g^−1^) by GCD					
0.2 A g^−1^	5.291	13.259	11.646	21.100	21.514
0.5 A g^−1^	2.365	8.742	7.994	14.911	17.086
1 A g^−1^	1.218	5.890	5.961	10.846	13.241
2 A g^−1^	0.728	3.159	3.271	6.862	10.226
5 A g^−1^	0.492	1.221	1.378	3.030	6.010
10 A g^−1^	0.380	0.664	0.770	1.631	3.684
EIS analysis					
Rs (Ω) (%error)	1.019 (0.542)	0.875 (0.474)	0.952 (1.422)	0.869 (1.596)	0.869 (1.657)
Rct (Ω) (%error)	0.793 (5.246)	0.474 (5.151)	0.419 (34.204)	0.274 (26.993)	0.217 (30.955)

The Nyquist plots of the pristine C12A7 and all C12A7_GO electrodes were studied by EIS measurements as shown in Fig. 12d. Normally, the Nyquist plots were obtained in two different frequency regions. At high frequency region, the intercept on Zʹ axis showed the series resistance (R_s_) of these electrodes and found to be 1.019, 0.875, 0.952, 0.869 and 0.869 Ω cm^2^ for the pristine C12A7, C12A7_GO-1, C12A7_GO-2, C12A7_GO-3 and C12A7_GO-4 electrodes, respectively. The semicircle loops of the Nyquist plots correspond to the charge transfer resistance (R_ct_) of the pristine C12A7 and all C12A7_GO electrodes, which can be determined from the diameter of a semicircle loop. Also, another semicircle at high frequency region was the result of diffusion process of 1 M KOH aqueous electrolyte. All determined R_s_ and R_ct_ values are summarized in Table 2. As seen in Table 2, the R_ct_ of the pristine C12A7 electrode was about 0.793 Ωcm^2^ indicating the imperfection diffusion process, whereas the R_ct_ values of C12A7_GO-1, C12A7_GO-2, C12A7_GO-3 and C12A7_GO-4 electrodes were lower than those of C12A7 electrode. The result was found to be 0.474, 0.419, 0.274 and 0.217 Ωcm^2^, for C12A7_GO-1, C12A7_GO-2, C12A7_GO-3 and C12A7_GO-4 electrodes, respectively. These outcomes suggested that the effective charge was transferred at the interface between electrode and electrolyte. Obviously, GO loading C12A7 cement electrode can be significantly enabled to more rapidly charge transfer at the C12A7_GO electrode and 1 M KOH aqueous electrolyte interface. At low frequency region, the slope of 45° portion of curve was due to Warburg resistance which represents the ion diffusion and/or transport in the electrolyte^49–53^. The capacitive behavior of the pristine C12A7 and all C12A7_GO electrodes was found to be good.

## Mechanical properties

**Figure 13:**
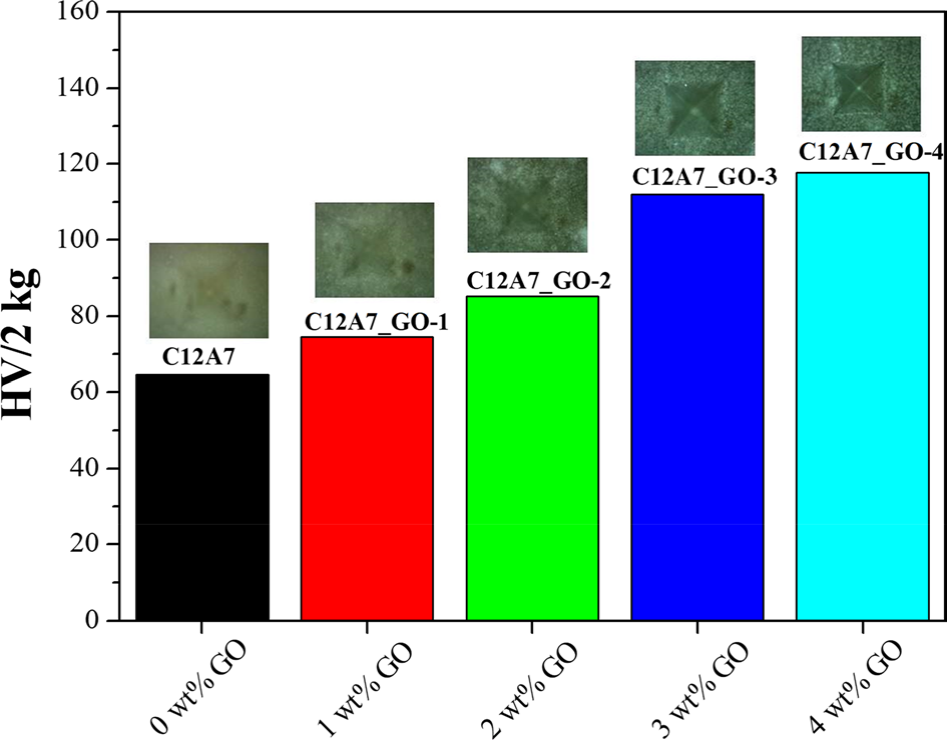
Micro-hardness of the pristine C12A7 and all C12A7_GO composite samples.

The mechanical properties of the pristine C12A7 and all C12A7_GO composites were investigated using a micro-hardness method with a pyramid on a square base according to the Vickers indenter hardness (*HV*) technique. The determined micro hardness defined the loading to the regions on the surface area by the following equation^26^:

5 $$HV = {1854}(P/{\text{d}}^{{2}} ),$$

where *HV* is the Vickers micro hardness value, *P* is the loading ratio in *kg,* and d is the average diagonal length of the impression in *mm*. The mechanical test applied 2 kg of a load weight for 5 s. As seen in Fig. 13, the *HV* values of the pristine C12A7, C12A7_GO-1, C12A7_GO-2, C12A7_GO-3 and C12A7_GO-4 composites were 64.6, 74.5, 85.2, 112.0, and 117.8 *H*V/2 kg, respectively. The results showed an increase in the HV values with increasing GO content. These results showed that the improvement of GO loading in pristine C12A7 cement was affected by the movement of free extra framework of O^2–^ directly between the cages through the cages opening^6,28,33^. Furthermore, these effects were due to the reinforcement of GO incorporation with an elastic force at grain boundary surface of C12A7 cement. This indicated that the C12A7_GO composite materials were affected by the stronger bonds between free extra framework of O^2–^ cement and free electrons on GO surfaces. It helped with inhibitive mechanism and the propagation and growth of initial microcracks^54^ as observed in SEM results (Fig. 6). Considering the high magnification view inset previously mentioned in SEM results, Fig. 6e suggests that the decrease of grain sizes of the C12A7_GO-4 composite was affected by loading weight percent of GO which has a high HV value. Thus, the enhancement of the mechanical properties of composited C12A7_GO cement specimens was affected by the amount of GO loading in pristine C12A7 cement.

## Conclusion

In summary, the C12A7_GO composites (C12A7_GO-1, C12A7_GO-2, C12A7_GO-3 and C12A7_GO-4) were successfully prepared by a direct mixing method of the pristine C12A7 and GO at room temperature. XRD result and Rietveld refinement method of pristine C12A7 and all C12A7_GO composites showed a pure cubic phase with a space group *Ia-3d* and the excellent fitting showed a good agreement with the 3CaO·Al_2_O_3_·6H_2_O standard. Raman spectroscopy confirmed the existence of GO in all C12A7_GO samples. Nitrogen sorption isotherms of all samples displayed a typical type-IV isotherm with the maximum specific BET surface area of 41.07 m^2^/g for C12A7_GO-4 samples. Scanning Electron Microscopy (SEM) showed the micrometer grain sizes of all samples and the occurrence of grain boundary interfaces for GO incorporation in all C12A7_GO samples. EDS also displayed a homogeneous distribution of the Ca, Al, O and C atoms on the powder surfaces. UV–Vis spectroscopy revealed that the absorption value of all C12A7_GO samples showed a red shift near longer wavelengths with increasing GO suspension loading. Interestingly, the E_g_ value of the pristine C12A7 sample was 5.427 eV, but the samples with GO loading C12A7 decreased from 5.364 to 5.288 eV with increasing the GO suspension loading. This was due to the red shift phenomenon in the UV absorption spectra originating from the formation of the free electrons interaction between free extra framework O^2–^ anions and GO surfaces in C12A7_GO lattices. Moreover, the dielectric constant of C12A7_GO composites can be explained by the high density of free electron charges resulting from the interfacial polarization on the GO surface. The electrochemical properties of the pristine C12A7 and all C12A7_GO composite electrodes displayed excellent capacitive properties as an indicator of the storage charge mechanism of electrical double layer capacitors (EDLCs) behavior. Interestingly, the maximum specific capacitance (*Csc*) of C12A7_GO-4 electrode was 21.514 at a current density of 0.2 A g^−1^. It is attributed to the increase in the electrochemically active surface area for the formation of the electrical double layer capacitors (EDLC) behavior and the effects of high surface area of GO connections. Besides, the *HV* values were increased with increasing GO to 64.6, 74.5, 85.2, 112.0, and 117.8 *H*V/2 kg of the pristine C12A7, C12A7_GO-1, C12A7_GO-2, C12A7_GO-3 and C12A7_GO-4 composites, respectively. The results indicated that the enhanced activity of the composite samples was due to an enrichment effect via diffusion within the modified electrode and highly useful GO surface area. Also, the free electron charge was transferred between the free extra framework O^2–^ anions and GO surfaces in C12A7_GO lattices. The C12A7_GO cement composites exhibited multifunctional properties appropriate for high-performance multifunctional applications such as smart cement materials, including optical, dielectric, electrochemical and mechanical properties. Future research may investigate the advantages of enhancing cement properties using modified surface cement from the 2-D nanostructure materials of the GO or Reduced Graphene oxide (rGO) suspension loading.

## Methods

### Chemicals

The raw materials were used to prepare the pristine C12A7 consisting of calcium carbonate (CaCO_3_, 99%) and alumina powder (Al_2_O_3_, 99.9%), which were purchased from Sigma Aldrich (USA). Acetone (99.9%, Lab grade high Quality) was obtained from Merck (Germany). All chemicals were used as received without further purification.

### Preparation of graphene oxide (GO) suspension

In this work, graphite oxide powder was synthesized via a modified Hummers method over oxidizing graphite with H_2_SO_4_ acid and KMnO_4_ oxidizing agent, as previously reported by Phrompet et al.^26^. Then, GO suspension was directly re-dispersed in acetone under ultra-sonication for 1.30 h followed by centrifugation at 8,000 rpm for 15 min. Finally, the dark brown supernatant of GO suspension at the concentration of 5 mg/mL was obtained.

### Preparations of C12A7 and all C12A7_GO composites

The pristine C12A7 was firstly prepared by a simple solid-state reaction based on Phrompet et al.’s^23^ study which reported the mixing of CaCO_3_ and Al_2_O_3_ powders in a mole ratio of 12:7. Then, the C12A7_GO composite samples were prepared using the pristine C12A7 mixed with various contents of GO suspension loading in 0, 1, 2, 3 and 4 wt.%, which was represented by the pristine C12A7, C12A7_GO-1, C12A7_GO-2, C12A7_GO-3 and C12A7_GO-4, respectively. In a typical procedure of C12A7_GO composite, about 10 g of the pristine C12A7 powder was dispersed with 150 mL of acetone under stirring. After it was vigorously stirred for 10 min, appropriate contents (1, 2, 3 and 4 wt%) of GO suspension were added and stirred for another 30 min. Then, the product samples were filtered and washed with acetone, followed by drying in oven at 80 ˚C overnight. Finally, all the C12A7_GO composite samples with the various contents of GO loading were obtained.

### Preparation of C12A7 and all C12A7_GO pellets

To measure dielectric properties, the powder of the pristine C12A7 and all C12A7_GO composite samples were constrained as pellets, which have the diameter pellets of 12 mm and a thickness of 2–3 mm using uniaxial compression at ~ 200 MPa. Then, the silver paste was painted on each pellet face of these samples. Next, all the obtained samples were heated at 150 °C for 2 h.

### Working electrodes assembly for electrochemical properties analysis

The analysis of electrochemical properties of working electrodes with the pristine C12A7 and all C12A7_GO composites was done in 1 M KOH aqueous electrolyte in three-system electrodes consisting of an active material (working electrode), silver/silver collide (Ag/AgCl reference electrode), and platinum wire (Pt counter electrode). According to Duangchuen et al.’ study^34^, the working electrodes were measured by Cyclic Voltammetry (CV test), Galvanostatic Charge–Discharge (GCD) test, and Electrochemical Impedance Spectroscopy (EIS) tests. The fabrication working cells for electrochemical characterization were normally prepared by the composition slurries mixed with a mass ratio of 80:10:10 of each obtaining product of polyvinylidene difluoride (PVDF) binder and acetylene black, respectively dissolved in N-methyl-2 pyrrolidone (NMP) of 0.4 ml. The composition slurries were mixed by using the ball milling method for 24 h. Next, the mixed composition was coated by a square area of 1 × 1 cm^2^ on a cleaned nickel foam substrate and subsequently dried at 80 °C for 6 h. Finally, all electrodes were pressed using uniaxial compression at 1.5 ton for 1 min and soaked in 1 M KOH aqueous electrolyte for 24 h before the test. The CV and GCD testing were carried out in the potential range of − 1.0 to 0.0 V at different scan rates of 10, 20, 50, 100 and 200 mV s^−1^ and different current density of 0.2, 0.5, 1, 2, 5 and 10 A g^−1^. The cycling stability testing of all working electrodes was determined using GCD test at a current density of 2 A g^−1^ for 1,000 cycles. Lastly, EIS testing of all working electrodes was carried out in a frequency range of 100 MHz–0.01 Hz using an open-circuit voltage by applying AC voltage of 10 mV.

### Characterizations

The structure of the pristine C12A7 and all C12A7_GO composites were investigated at 2θ scanning range of 5–85° with a step interval of 0.02°/s by X-Ray Diffractometer (XRD) in Rigaku (Miniflex Cu K-alpha radiation (CuKα) = 1.5406 Å). Moreover, the confirmed characteristic structures of the pristine C12A7 and the interaction interface between GO and pristine C12A7 of all C12A7_GO composites were examined using dispersive Raman microscopy DXR Smart (Thermo Scientific) at a 532 nm excitation wavelength. Also, FTIR spectra (Bruker, Senterra) was used to evaluate the vibration mode of the atomic bonding of all samples. Thermogravimetric Analysis (TGA) and Netzsch STA 449F3 Jupiter were performed over 35–900 °C with a heating rate of 10 °C/min in nitrogen gas to measure the weight loss of all samples. Pore-size distribution and specific BET surface area of all samples were studied from the Nitrogen gas adsorption/desorption isotherm using the BET and Barrett-Joyner-Halenda (BJH) technique in Autosorb-1, Quantachrome. Scanning Electron Microscope (JEOL SEM JSM-5800 LV) was used to observe the morphologies and grain sizes of the pristine C12A7 and all C12A7_GO composites and to perform the homogeneous distribution of atoms on the powder surfaces by energy dispersive X-ray spectroscopy (EDX-mapping). Shimadzu UV-3101PC UV–Vis–NIR spectrophotometer was utilized to measure the absorption edge spectra of all samples at room temperature based on Rudradawong et al.’s study^21^. Furthermore, the dielectric properties of all samples at room temperature were investigated within an AC oscillation voltage of 500 mV over the frequency of 40–10^7^ Hz in a KEYSIGHT E4990A Impedance Analyzer. Finally, the electrochemical workstation (CS350 Potentiostat/Galvanostat, Wuhan Corrtest Instruments Corp Ltd) conducted the investigation on electrochemical properties of working pristine C12A7 and all C12A7_GO composite electrodes.
